# Plant regeneration through somatic embryogenesis in cell suspensions of *Cenchrus ciliaris* L.

**DOI:** 10.1186/s13007-023-01081-3

**Published:** 2023-10-18

**Authors:** Shipra Goyal, Vijaya Chatterjee, Vishvas M. Kulkarni, Vishnu Bhat

**Affiliations:** 1 Plant Developmental Biology, Department of Botany, University of Delhi, Delhi, 110007 India; 2 Nuclear Agriculture and Biotechnology Division, Bhabha Atomic Research Centre, Mumbai, 400085 India; 3 Department of Biotechnology, St. Aloysius College, Jabalpur, 482001 India

**Keywords:** Cell suspensions, Cell growth curves, Somatic embryogenesis, In-vitro plant regeneration, *Cenchrus ciliaris*

## Abstract

**Background:**

This study aims to establish cell suspension culture, its maintenance and induction of somatic embryogenesis, and in vitro plant regeneration in *Cenchrus ciliaris* L. Suspension cultures are relatively homogenous cell lines facilitating uniform access to nutrition. These are ideal sources of competent cells for genetic transformation.

**Results:**

Callus was initiated by culturing immature inflorescences of *Cenchrus ciliaris* cv. IGFRI-3108 on Murashige and Skoog (MS) medium containing 3 mg l^−1^ 2,4-dichlorophenoxyacetic acid (2,4-D) and 0.5 mg l^−1^ 6-benzylaminopurine (BAP) with 30 g l^−1^ sucrose. Cell suspension cultures were established in liquid MS medium using an inoculum size of 10 g l^−1^. These were maintained to achieve embryogenic cell/regeneration competent cultures. Growth curve analysis and a subculture interval of 20 days were determined to harvest cells at the end of the exponential phase. The cell doubling time was found to be 11 days. Somatic embryogenesis was accomplished in MS medium containing 1 mg l^−1^ 2,4-D, 1 mg l^−1^ BAP along with growth adjuvants, 300 mg l^−1^ casein hydrolysate, 400 mg l^−1^ glutamine and 300 mg l^−1^ proline. The highest number (16 ± 3.78/per inoculum) of shoots regenerated on this medium. The elongation and rooting of shoots were recorded on basal MS and ½ MS media, respectively. Rooted plants were successfully transferred to pots containing a Soilrite and cocopeat mixture in a 3:1 proportion for 3–4 weeks and later successfully acclimatized in the greenhouse with a 60% survival rate. The genetic fidelity of 12 regenerated plants was analysed using RAPD primers that were genetically identical to the mother plant.

**Conclusion:**

Cell suspension culture-based in vitro plant regeneration of *C. ciliaris* involved the establishment, maintenance and progression of somatic embryogenesis during shoot and root development. The inherent limitation of callus-mediated in vitro plant regeneration reducing the regeneration potential due to the aging of the calli has been overcome.

## Background/introduction

Callus derived from seeds and immature inflorescences of Buffelgrass (*Cenchrus ciliaris* L.) are affected by genotypes and exhibit highly varied regeneration potential [1, 2]. Several attempts have been made towards plant regeneration through direct and indirect shoot organogenesis using several explants [1, 3]. Shekhawat et al. [4] also reported better explants for the establishment of competent/regenerative cultures in Pearl millet (*Pennisetum glaucum* (L.) R. Br. Syn. *Cenchrus americanus* (L.) as also reported by [5]. However, optimization of the genetic transformation of *C. ciliaris* using regenerative calli has achieved limited success [6–8]. Transgenic shoots were raised from the bombarded calli. These did not root [8]. *Cenchrus ciliaris* is recalcitrant to *Agrobacterium*-mediated genetic transformation [8, 9].

Cell suspensions are useful for both cell biological research and genetic transformation in grasses [10–15]. Homogeneous cell suspensions as targets of transformation may increase the frequency of transgenic events, as the advanced age of calli hinders the regeneration frequency of transgenic calli [16]. Homogenous cell suspensions facilitate uniform access to nutrition precursors and growth hormones [17]. Moreover, additional information was generated during the cell suspension culture study on inoculum size, cell doubling time and subculture interval. This information can be used effectively to decide the critical stage of competent suspension cells to be used for genetic transformation [18].

The highly recalcitrant nature of transgenic embryogenic calli for regeneration can be overcome by experimentally manipulating the rate of cell multiplication. The establishment of cell suspension-derived plant regeneration helps in studying the response of cell suspensions to supplementation with various hormones, growth adjuvants and osmotic stress. Similarly, it is possible to determine a more accurate stage of transfer of cell suspensions for inducing somatic embryos that eventually develop shoots and roots [19]. Regenerable cell suspensions developed during this study provide ideal sources of competent cells for the genetic transformation of *C. ciliaris.*

## Materials and methods

### Callus induction and initiation of suspension cultures

Immature inflorescences at stage IV [8] were collected from the cultivar IGFRI-3108 for callus induction. Inflorescences were surface sterilized in 70% ethanol for 3 min and repeatedly washed 3–4 times with autoclaved distilled water. Immature inflorescences were dissected into 1 cm long pieces (approx.) and cultured under aseptic conditions on MS containing 3 mg l^−1^ 2,4-D and 0.5 mg l^−1^ 6 BAP following [8]. Eight different MS suspension media combinations were used with different 2,4-D and BAP concentrations. Only friable calli were selected to initiate cell suspension cultures from the calli. Callus induction from different parts of an explant (i.e., immature inflorescence) was considered as different cell lines. Ten different cell lines were used to monitor each single hormone composition. MS medium was used as a control. Calli were suspended in 250 ml Erlenmeyer flasks containing 30 ml media each for all compositions, including the control. Suspensions were incubated in a gyratory shaker at 100 RPM and 27 °C in the dark. The medium was replaced every seven days by pipetting out 75% of the old media from cultures and adding fresh medium to the same. After the completion of one growth cycle, cells were subcultured by making 4–5 replicates from single cell line growth.

### Determination of inoculum weight for cell suspensions

Inoculum weight for initiation of cell suspensions was optimized at 6 levels viz; 5 g l^−1^ (150 mg/30 ml), 10 g l^−1^ (300 mg/30 ml), 15 g l^−1^ (450 mg/30 ml), 20 g l^−1^ (600 mg/30 ml), 25 g l^−1^ (750 mg/30 ml), and 30 g l^−1^ (900 mg/30 ml). All inoculums were suspended in liquid MS with 3 mg/l 2,4-D, 0.5 mg l^−1^ BAP and 30 g l^−1^ sucrose. After the completion of one growth cycle, cells were filtered using autoclaved Whatman filter paper (150 mm circle), and cells were collected over the filter paper. Cell aggregates were transferred to autoclaved and pre-weighed 50 ml capacity Falcon tubes. The fresh weight of cells was recorded at the end of the growth cycle for all six inoculums. Cells were dried at 37 °C overnight in a hot air oven, and dry weights were recorded repeatedly until the weight readings became constant. The growth rate (%) was calculated using inoculum weight and final fresh weight data.

$${\text{Growth rate }}\left( \% \right) = \frac{{\left( {{\text{Final fresh weight}} - {\text{Inoculum weight}}} \right) \, \times { 1}00}}{{\text{Inoculum weight}}}$$

Cells were pelleted down by centrifugation at 1000 RPM for 30 s, and the packed cell volume (PCV) was calculated.

$${\text{PCV}}\left( \% \right) = \frac{{{\text{Volume of packed cells }} \times { 1}00}}{{{\text{Total volume}}\left( {{\text{cells}} + {\text{media}}} \right)}}$$

### Growth dynamics of cell suspension cultures

The growth pattern of cells in suspension was recorded every 5 days for 1 month, and a growth curve was plotted. Only 3 inoculums were selected based on high growth rate (%) and PCV (%) for this study, i.e., 5 g l^−1^, 10 g l^−1^, and 15 g l^−1^. Ten microliters of culture was pipetted out and dropped in a microcentrifuge tube with 10 µl of 0.4% trypan blue and mixed well by pipetting twice. Immediately, a total of 20 µl of culture was loaded on a hemocytometer (Neubauer improved, Germany), and cells were counted under a light microscope (Zeiss). Cells were counted in all 4 quadrants separately, and an average number of cells was considered as the final reading. Cells that were darkly stained with dye and showed shrunken nuclei were considered dead, and light-stained cells were considered alive. This was repeated three times for all three inoculums, and the average numbers of total, dead and live cells were calculated. Finally, to calculate cells present in 1 ml, a formula was applied, i.e., Cells ml^−1^ = No. of cells x dilution factor × 10^4^ (dilution factor was taken as 2 because cell culture and trypan blue were mixed in a 1:1 ratio).

### Scanning electron microscopy

Different developmental stages of somatic embryos from suspension-mediated calli were identified under a stereomicroscope (KL 200, ZEISS) and fixed in 2% glutaraldehyde. After overnight fixation, the samples were processed through an acetone series (10%, 30%, 50%, and 70%). Samples were stored only in 70% acetone before final imaging. One day before imaging, they were processed in 80%, 90% and 100% acetone at 4 °C and dried using the critical point drying (CPD) method. Then, the samples were mounted on aluminium stabs coated with silver tape for imaging. After mounting, imaging was processed on a scanning electron microscope (EVO LS10, Carl Zeiss, Germany).

### Somatic embryogenesis and plantlet regeneration from cell suspensions

Cells from the exponential growth phase were used for experiments. The cells were passed through Whatman filter paper (150 mm circles). For regeneration, a total of 17 hormone combinations (using 6-BAP, 2,4-D, thiadiazuron (TDZ), kinetin, indole 3-acetic acid (IAA) and selected amino acids) were used, including basal MS as control media. Media were poured into 250 ml flasks. Five to six small cell aggregates were plated on regeneration media in each flask. A total of 20 cell groups were plated in each combination and incubated in a room with a 16-h photoperiod at 25 °C. Once the shoots came into sight, they were transferred to basal MS for further elongation and development.

### Root development and acclimatization of plants

Plantlets were transferred for rooting in different MS media combinations with and without exogenous auxin supply. MS media with 2 mg l^−1^ indole-3-butyric acid (IBA), MS with 2 mg l^−1^ IBA and 0.4% charcoal, ½ MS without any growth regulator and basal MS as control media were used to check root growth and development. Gelrite (2 g l^−1^) was used as a gelling agent with 30 g l^−1^ sucrose in rooting media.

Rooted plants were washed with distilled water to remove sucrose particles. Plants were kept on blotting sheets and dried for some time. Autoclaved soil rite and cocopeat were mixed in a 3:1 proportion using Hoagland solution. Thermocol cups (diameter 6.6 cm) were filled with prepared soil, and plants were planted carefully in those cups. A humidity chamber was created for plants by covering them using NaturalWrap cling film by making some holes in it for required aeration. After some days, plants were transferred to pots containing garden soil and kept in greenhouse conditions. Sufficient water and nutrient supplies were provided to the plants at intervals of 2–3 days. After one month of successful adaptation of plants in the greenhouse, the pots were transferred to the open field for acclimatization.

### Analysis of genetic similarity with the mother plant

Twelve in vitro regenerated plants were PCR analysed using RAPD primers to check the extent of genetic similarity. Genomic DNA was isolated from leaves using the cetyltrimethylammonium bromide (CTAB) method [20]. The DNA concentration was estimated using Multiskan GO (Thermo Scientific). DNA samples were diluted, and the concentration was adjusted up to 50 ng µl^−1^. A 25 µl reaction mix was prepared for each DNA sample using 10 × PCR buffer, MgCl_2_ (25 mM), dNTPs (10 mM), primers (10 mM), Taq polymerase (BR Biochem) (0.25 unit/rxn.), autoclaved ddH_2_O and DNA (1 µl of 50 ng µl^−1^) as a template. PCR tubes were arranged in My Cycler (Bio-Rad). Reaction conditions were set as denaturation at 94 °C for 5 min (for the 1st cycle), [94 °C for 1 min, annealing at 37 °C for 1 min, extension at 72 °C for 3 min] for 30 cycles, final extension at 72 °C for 10 min and hold at 4 °C for infinite. Gel electrophoresis was performed for the PCR products, and gel pictures were captured using a BioDoc-It™ Imaging System (UVP). Total bands were scored by counting manually.

### Data analysis

Data on fresh weight, growth rate (%), dry weight and PCV (%) were recorded and estimated at an interval of five days for each inoculum. Dead cells and live cells were counted every 5 days, and curves were plotted using the same method. Data for somatic embryo formation and plantlet regeneration were collected from 20 cell groups for each hormonal treatment on alternate weeks. Total root development data were collected from a group of three plants for each hormonal treatment at the time of hardening. Root growth in ½ MS was observed, and data were collected every 5 days. All experiments were repeated three times. Data were analysed using IBM SPSS Statistics 21 software. One-way ANOVA was performed with a significance level of p ≤ 0.05. Duncan’s multiple range test (DMRT) was also performed to test the significant differences between the mean values of the same variable at p ≤ 0.05.

## Results

**Fig. 1:**
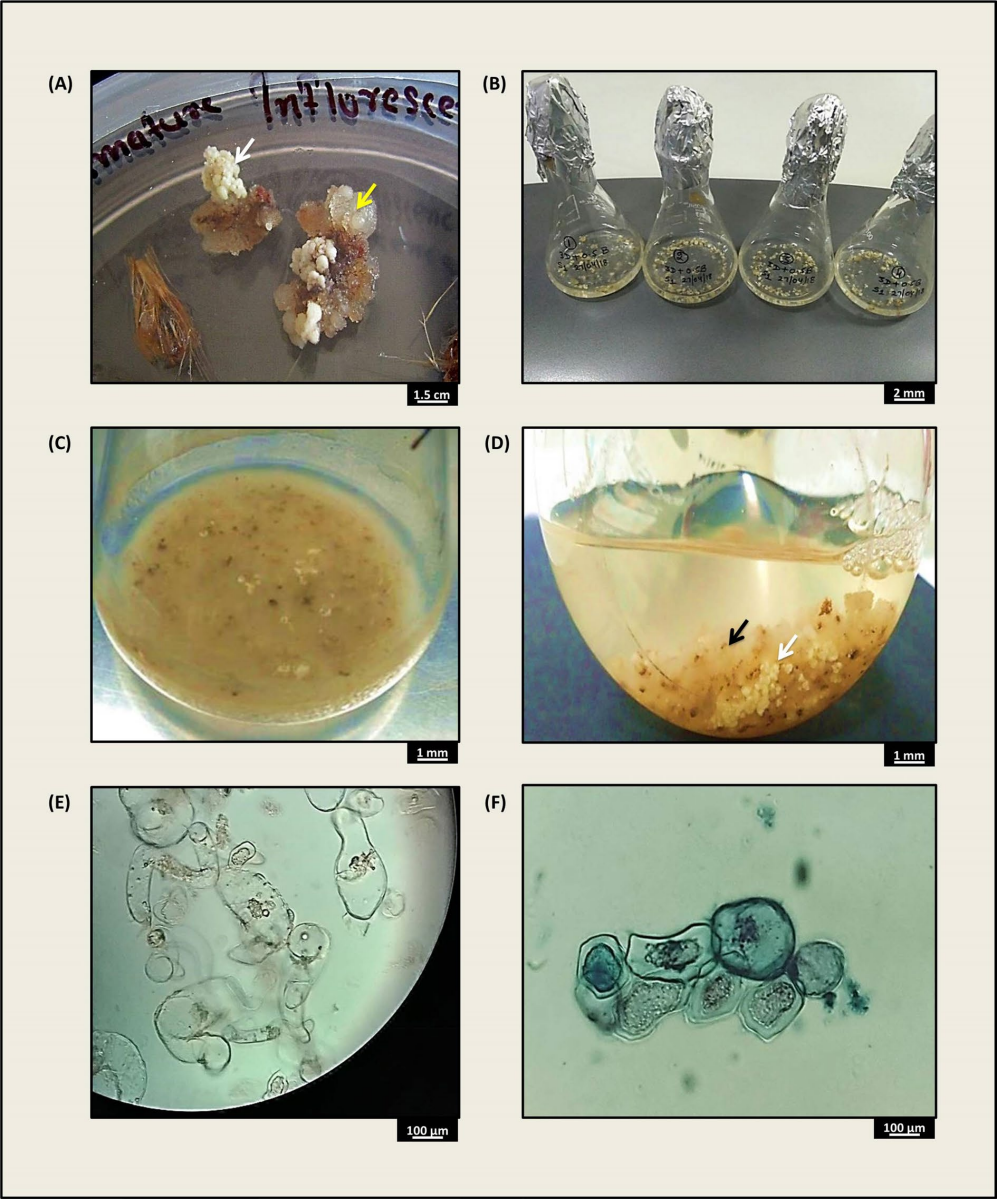
Cell suspension initiation and different phases of cell growth in a suspension medium, **A** induced white friable callus (white arrow) and loose, watery callus (yellow arrow), **B** batches of suspensions initiated using white friable callus, **C** growth observed after 20 days, **D** embryogenic (white arrow) and non-embryogenic (black arrow) cells grown in a suspension medium, **E** proliferating cells, **F** light stained (live) and dark stained (dead) cells with trypan blue

Immature inflorescence-derived cell suspensions (Fig. 1A–D) were used for MS media optimization for suspension initiation, inoculum optimization for maximum growth rate, cell growth (Fig. 1E), and live and dead cell identification (Fig. 1F). The results of the study are presented herewith.

### Optimization of cell growth with plant growth regulators

**Fig. 2:**
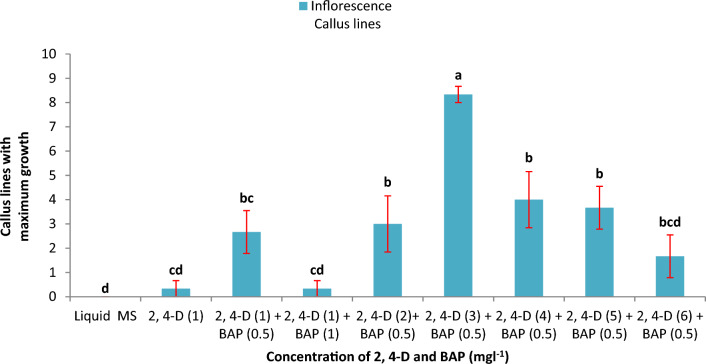
Effect of plant growth regulator concentration on cell suspension growth. Different letters in lowercase represent significant differences at p ≤ 0.05 according to Duncan’s multiple range test (DMRT)

The highest number of healthy cell lines resulted in maximum growth with 3 mg l^−1^ 2,4-D and 0.5 mg l^−1^ BAP. A high concentration of 2,4-D was growth-limiting for cells (Fig. 2). Cells with high 2,4-D concentrations were brown and were unable to reach the maximum growth rate. At lower 2,4-D concentrations, proliferation was very slow, and more browning of the cell groups was observed. Liquid MS lacking plant growth regulators was unable to support cell division, and all cell lines were found to brown. BAP (0.5 mg l^−1^) was added with 2,4-D because 2,4-D alone was insufficient for cells to initiate proliferation. A high BAP (1 mg l^−1^) concentration was found to be growth-limiting for the cells during cell suspension growth (Fig. 2).

### Inoculum for suspension medium in optimized PGR concentration

The fresh weight after one complete cycle of cell culture was the highest, i.e., 89.99 ± 1.89 (g l^−1^) recorded with 10 g l^−1^ inoculum. The dry weight, i.e., 9.86 ± 0.26 (g l^−1^), and PCV %, i.e., 11.67 ± 1.20, were also highest for the 10 g l^−1^ inoculum. The growth rate was 799.99 ± 18.95 with 10 g l^−1^ inoculum. When the inoculum was small, i.e., 5 g l^−1^, the growth rate was very slow, PCV was lowest and cells were little brown in appearance after one growth cycle. As the inoculum size increased, the dynamic growth rate increased up to 800% (in 10 g l^−1^ inoculum) and 485% (in 15 g l^−1^ inoculum) (Table 1). PCV was also highest for the two inoculums after the completion of one successful growth cycle. Beyond that, all inoculum sizes were observed to have a poor cell growth rate and more browning in the culture.

**Table 1:** Effect of inoculums on cell suspension growth in liquid MS with 3 mg l^−1^ 2,4-D and 0.5 mg l^−1^ BAP

S. no.	Inoculum (g l^−1^)	Final fresh weight (g l^−1^)	Growth rate (%)	Dry weight (g l^−1^)	Packed cell volume (%)
1	5	16.22 ± 0.44^e^	224.35 ± 8.85^c^	1.78 ± 0.16^e^	1.10 ± 0.21^d^
2	10	89.99 ± 1.89^**a**^	799.99 ± 18.95^**a**^	9.86 ± 0.26^**a**^	11.67 ± 1.20^**a**^
3	15	87.78 ± 0.78^a^	485.18 ± 5.19^b^	9.72 ± 0.24^a^	8.63 ± 0.33^b^
4	20	64.44 ± 1.25^b^	222.20 ± 6.26^c^	7.01 ± 0.23^b^	4.90 ± 0.58^c^
5	25	54.77 ± 0.29^c^	119.09 ± 1.18^d^	5.97 ± 0.27^c^	2.70 ± 0.47^d^
6	30	47.33 ± 0.51^d^	57.77 ± 1.69^e^	5.17 ± 0.26^d^	1.86 ± 0.15^d^

A total of 3 flasks were considered as one event for each reading and every inoculum

Mean ± SE followed by different letters in superscript for a column is significantly different at P ≤ 0.05 according to Duncan’s multiple range test (DMRT)

### Effect of inoculum size on cell growth rate and dynamic growth pattern

**Fig. 3:**
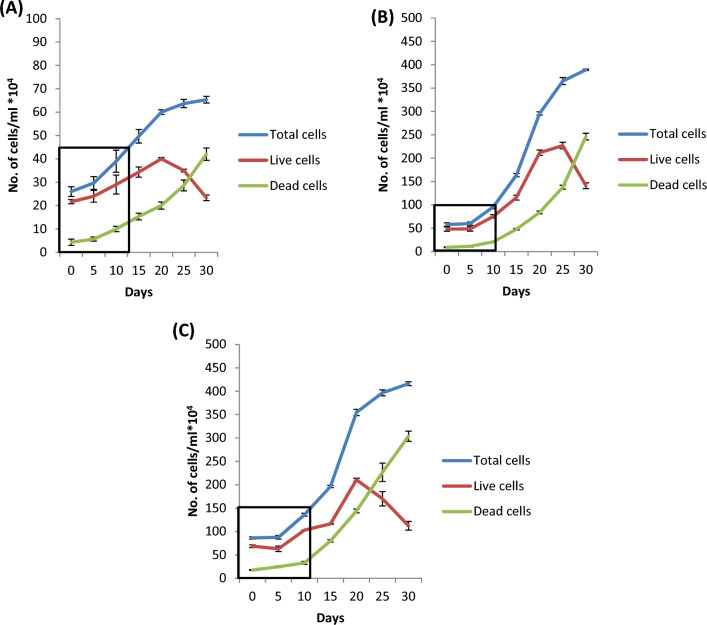
Cell growth curves for the culture duration, **A** Curve plotted for inoculum 5 g l^−1^ using total cells, live cells and dead cells data, **B** Curve plotted for inoculum 10 g l^−1^ using total cells, live cells and dead cells data, **C** Curve plotted for inoculum 15 g l^−1^ using total cells, live cells and dead cells data. Data were analysed by Duncan’s multiple range test (DMRT) and found to be significantly different at p ≤ 0.05

Three inoculum sizes with the highest fresh weight and growth rate (Table 1) were selected for detailed analysis of cell proliferation and cell doubling time optimization with a maximum number of live cells (Fig. 1E and F). For inoculum 5 g l^−1^, the initial cell counts for total cells, live cells and dead cells were 23 × 10^4^ cells ml^−1^, 20 × 10^4^ cells ml^−1^ and 3 × 10^4^ cells ml^−1^, respectively, and the final cell counts were 65.34 × 10^4^ cells ml^−1^, 23.33 × 10^4^ cells ml^−1^ and 42 × 10^4^ cells ml^−1^, respectively. The initial cell counts for total cells, live cells and dead cells in inoculum 10 g l^−1^ were 51.50 × 10^4^ cells ml^−1^, 40.49 × 10^4^ cells ml^−1^ and 11.1 × 10^4^ cells ml^−1^, respectively, and the final cell counts were 389.31 × 10^4^ cells ml^−1^, 141 × 10^4^ cells ml^−1^ and 246.35 × 10^4^ cells ml^−1^, respectively. Inoculum 15 g l^−1^ initially had total cells, live cells and dead cells counts of 82 × 10^4^ cells ml^−1^, 63 × 10^4^ cells ml^−1^ and 19 × 10^4^ cells ml^−1^ and final cell counts of 416 × 10^4^ cells ml^−1^, 112.33 × 10^4^ cells ml^−1^ and 303.67 × 10^4^ cells ml^−1^, respectively (Fig. 3). The cell doubling times were 13 days (Fig. 3A), 11 days (Fig. 3B) and 12 days (Fig. 3C) for the 5 g l^−1^, 10 g l^−1^ and 15 g l^−1^ inocula, respectively. The fastest growth was observed in the 10 g l^−1^ culture inoculum. Live cells in the culture were present for the longest duration, i.e., 27 days, in the 10 g l^−1^ inoculum culture, while in the other two cultures, the number and duration of viable cells were lower (Fig. 3).

### Stages of somatic embryo development

**Fig. 4:**
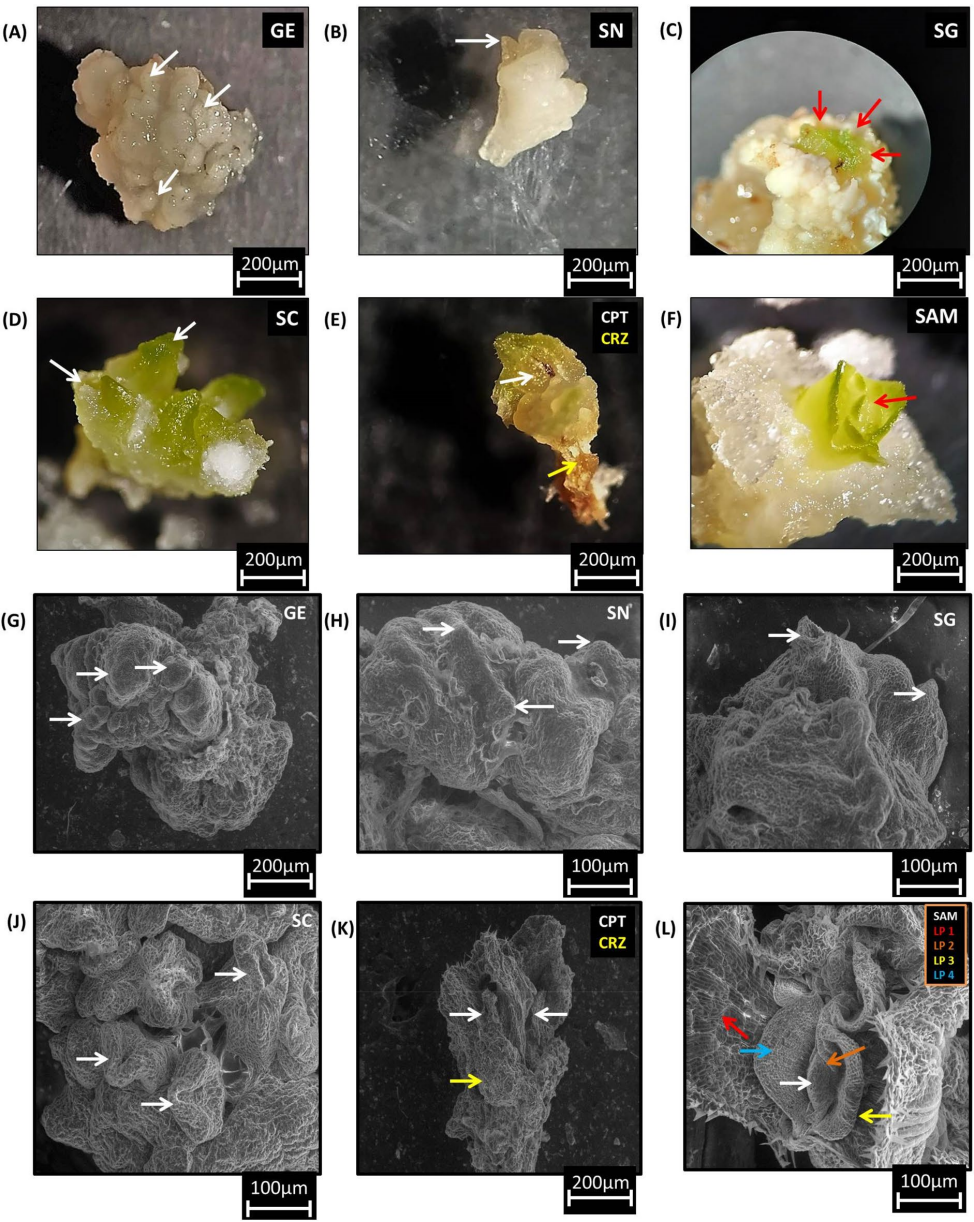
**A**–**F** Identified and isolated somatic embryo development stages. **A** Globular embryo formation, **B** scutellar notch, **C** and **D** advance scutellar stages, **E** coleoptile (CPT) and coleorhiza (CRZ), **F** leaf primordia development stage, **G**–**L** scanning electron micrographs of different stages of somatic embryos from cell suspension cultures (**G**) globular embryo stage, **H** globular embryo differentiation in the scutellar notch (SN), **I**)scutellar grooves (SG) formation, **J** differentiated scutellum (SC), **K** arrows indicating coleoptile (CPT) and coleorhiza (CRZ) followed by scutellum, **L** shoot apical meristem (SAM) surrounded with leaf primordia (LP 1, LP 2, LP 3, LP 4) layers in the process of leaf formation

Plant growth regulators were optimized for somatic embryo development (Table 2). Somatic embryo development from cell suspensions was captured under a stereomicroscope from the globular embryo stage (Fig. 4A–F). In scanning electron micrographs, the globular embryo stage was identified from 3-day-old plated cells on regeneration media (Fig. 4G). Scutellar notches were observed on the cell groups after 5 days of plating (Fig. 4H). Scutellar groove formation was noticed in scutellar structures after 6 to 7 days of plating (Fig. 4I). Complete scutellum was spotted after 10 days of culturing (Fig. 4J). Coleoptiles were observed under the scutellum when two-week-old plated cultures were fixed and screened (Fig. 4K). Once greening appeared on plated cultures, embryos were dissected out under a stereomicroscope and fixed in a fixative. Scanning electron micrographs were obtained to reveal the leaf primordia arrangement around the shoot apical meristem (SAM) (Fig. 4L).

**Table 2:** Shoot induction in different growth hormone combinations

Media combination								Response		
S. no.	MS	BAP mg l^−1^	2,4-D mg l^−1^	TDZ mg l^−1^	IAA mg l^−1^	Kinetin mg l^−1^	Amino acids	Total no. of embryos formed	Total no. of shoots formed after 3 weeks	Total no. of shoots formed after 5 weeks
1	Basal MS	–	–	–	–	–	–	–	–	–
2	Basal MS	1	0.5	–	–	–	–	26.33 ± 2.18^de^	1.00 ± 0.00^c^	2.34 ± 1.20^c^
3	Basal MS	1	0.5	–	–	–	CGP*	39.31 ± 2.84^c^	4.00 ± 1.15^b^	9.33 ± 0.88^b^
4	Basal MS	2	0.5	–	–	–	–	14.00 ± 2.08^f^	–	–
5	Basal MS	3	0.5	–	–	–	–	6.66 ± 0.88^gh^	–	–
6	Basal MS	4	0.5	–	–	–	–	–	–	–
7	Basal MS	5	0.5	–	–	–	–	–	–	–
8	Basal MS	1	1	–	–	–	CGP*	66.00 ± 4.04^a^	8.00 ± 1.15^a^	16.00 ± 3.78^a^
9	Basal MS	1	–	0.1	1	–	–	20.62 ± 1.45^e^	–	–
10	Basal MS	1	–	0.1	–	–	–	25.34 ± 2.72^de^	–	–
11	Basal MS	1	–	–	–	–	–	–	–	–
12	Basal MS	0.1	–	0.1		–	–	31.00 ± 2.30^d^	–	2.00 ± 1.15^c^
13	Basal MS	0.1	–	0.1	1	–	–	50.65 ± 4.40^b^	3.00 ± 0.58^b^	11.00 ± 2.30^b^
14	Basal MS	3	0.25	–	1	–	–	21.29 ± 3.75^e^	–	–
15	Basal MS	3	0.25	–	–	0.1	–	11.00 ± 1.73^fg^	–	–
16	Basal MS	3	–	–	1	1	–	8.66 ± 1.45^fg^	–	–
17	Basal MS	–	–	–	1	0.1	–	–	–	–

CGP* casein hydrolysate, glutamine and proline

A total of 20 cell groups were used each time for regeneration in each media combination

Mean ± SE followed by different letters in superscript for a column are significantly different at P ≤ 0.05 according to Duncan’s multiple range test (DMRT)

### Effect of PGRs and amino acids on shoot induction

**Fig. 5:**
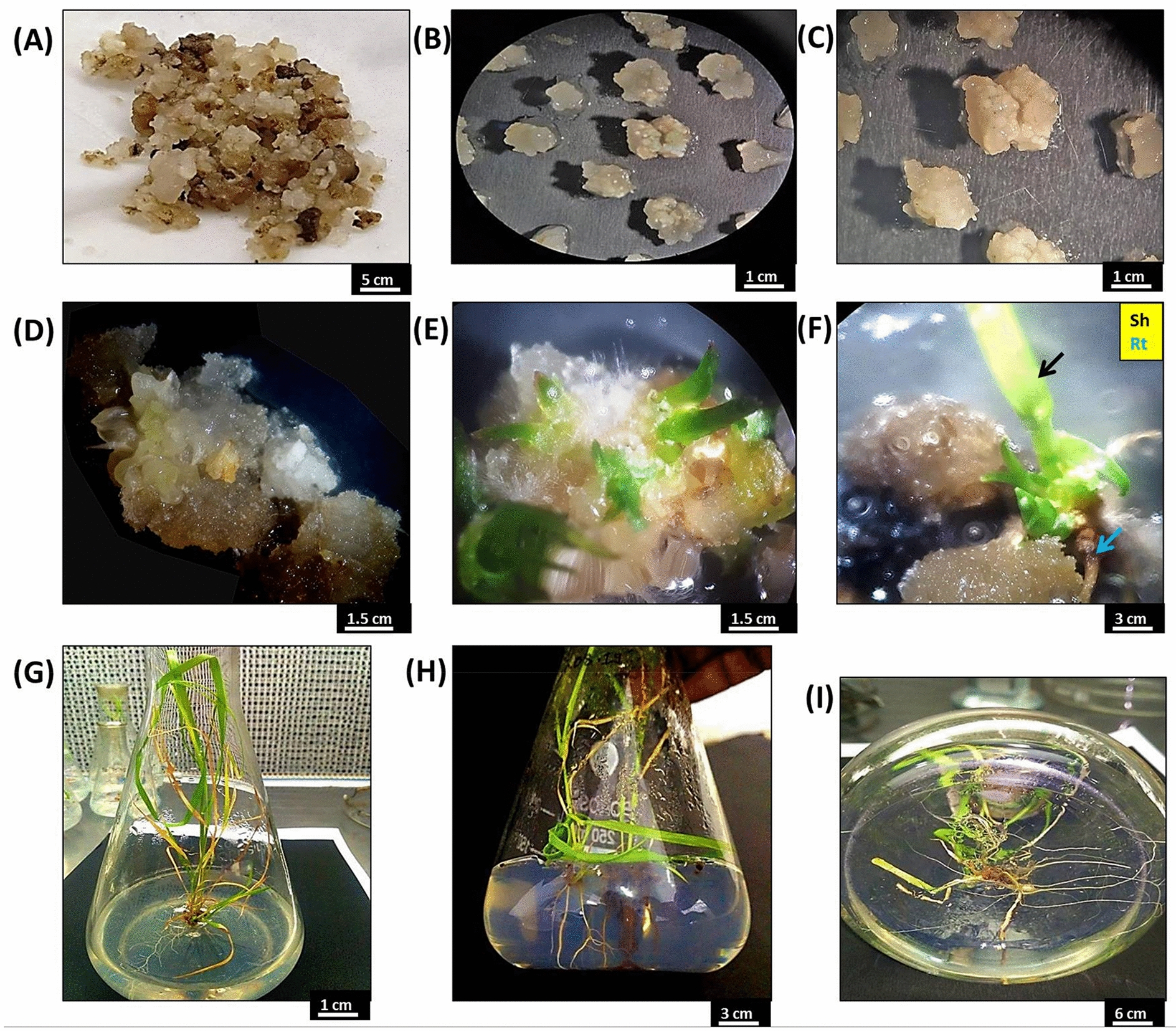
Regenerating suspension cell groups, **A** filtered cells collected on Whatman filter paper, **B** and **C** isolated white and small cell groups plated on regeneration medium, **D** somatic embryogenesis (**E**) shoot growth, and **F** shoot (Sh) and root (Rt) on MS with plant growth regulators, **G** developing plants on basal MS, **H** complete in vitro regenerated plants, **I** root growth on ½ MS

Many plant growth regulators were used in different combinations to optimize somatic embryogenesis and shoot development from cell suspensions. Somatic embryogenesis in cell suspensions could take place in most of the media combinations provided (Table 2), but successful shoot development could not be accomplished in all media combinations. The highest number of embryo formations could be observed in 1 mg l^−1^ BAP, 1 mg l^−1^ 2,4-D with 300 mg l^−1^ casein hydrolysate, 400 mg l^−1^ l-glutamine and 400 mg l^−1^ l-proline, i.e., 66.00 ± 4.04 (Table 2). When the 2,4-D concentration was reduced to half (0.5 mg l^−1^), the embryo formation rate declined, i.e., 39.31 ± 2.84. When TDZ (0.1 mg l^−1^) and IAA (1 mg l^−1^) were added along with a low BAP concentration (0.1 mg l^−1^), the rate of embryo formation increased up to 50.65 ± 4.40. Shoot development results were also similar, i.e., 16.00 ± 3.78, 9.33 ± 0.88 and 11.00 ± 2.30, respectively, in the abovementioned PGR combinations (Table 2). All other hormone combinations did not promote shoots from the somatic embryos. From plating up to shoot formation (Fig. 5A–E), it was a complete 5–7 week process, and once shoots and roots appeared, they were transferred to basal MS for further growth and elongation (Fig. 5F–I).

### Root growth and acclimatization of plantlets

**Fig. 6:**
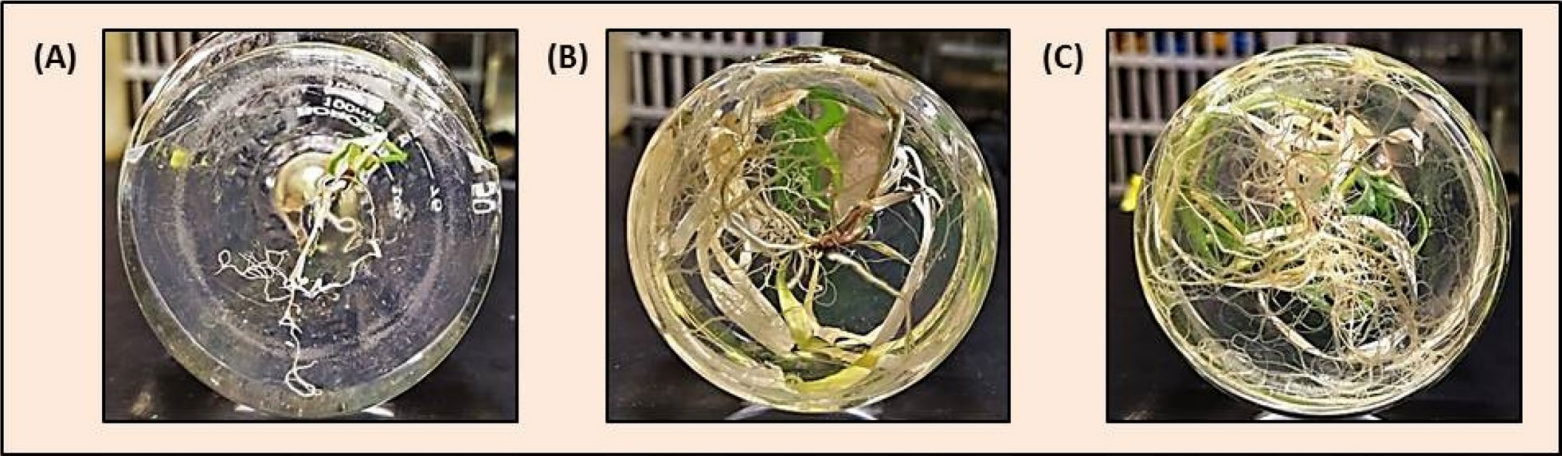
Root growth in ½ MS, **A** after 7 days, **B** after 15 days, **C** after 25 days

Root growth was observed on basal MS and ½ MS without an external auxin supply. However, indole-3-butyric acid (IBA) was also used in different concentrations with or without 0.4% charcoal, but rooting could not improve (Table 3). White and healthy roots could be observed on basal MS, but ½ MS was a better medium for thick and long roots. Root growth was optimized in ½ MS, and data were recorded at 5-day intervals for 30 days (Fig. 6A–C). A significant increase in root number and root length was observed in ½ MS in comparison to MS with IBA and basal MS (Table 4).

**Table 3:** Root growth in ½ MS, basal MS and different IBA concentrations

S. no.	Media composition	No. of roots	Root length (cm)	Observation
1	½ MS	29.33 ± 1.45^a>^	10.90 ± 0.83^a^	White, thick and long roots
2	Basal MS	3.33 ± 0.33^b^	1.10 ± 0.23^b^	White, small threads like roots
3	MS + 1 mg l^−1^ IBA	1.33 ± 0.29^b^	0.40 ± 0.15^b^	Small and brown roots
4	MS + 2 mg l^−1^ IBA	2.67 ± 0.38^b^	0.47 ± 0.18^b^	Small and brown roots
5	MS + 2 mg l^−1^ IBA + 0.4% Charcoal	1.00 ± 0.28^b^	0.17 ± 0.04^b^	Small and brown roots

The number of roots and root length were calculated by taking an average of 3 plant data points

Mean ± SE followed by different letters in superscript for a column is significantly different at P ≤ 0.05 according to Duncan’s multiple range test (DMRT)

**Table 4:** Root growth with days in ½ MS

S. no.	Data recorded after (days)	No. of roots	Root length (cm)
1	5	2.33 ± 0.33^f^	0.27 ± 0.07^f^
2	10	5.00 ± 0.57^e^	1.87 ± 0.19^e^
3	15	8.67 ± 0.88^d^	4.77 ± 0.20^d^
4	20	15.67 ± 0.88^c^	6.63 ± 0.17^c^
5	25	22.00 ± 0.57^b^	8.80 ± 0.58^b^
6	30	29.33 ± 1.45^a^	10.90 ± 0.83^a^

The number of roots and root length were calculated by taking an average of 3 plant data points

Mean ± SE followed by different letters in superscript for a column are significantly different at P ≤ 0.05 according to Duncan’s multiple range test (DMRT)

**Fig. 7:**
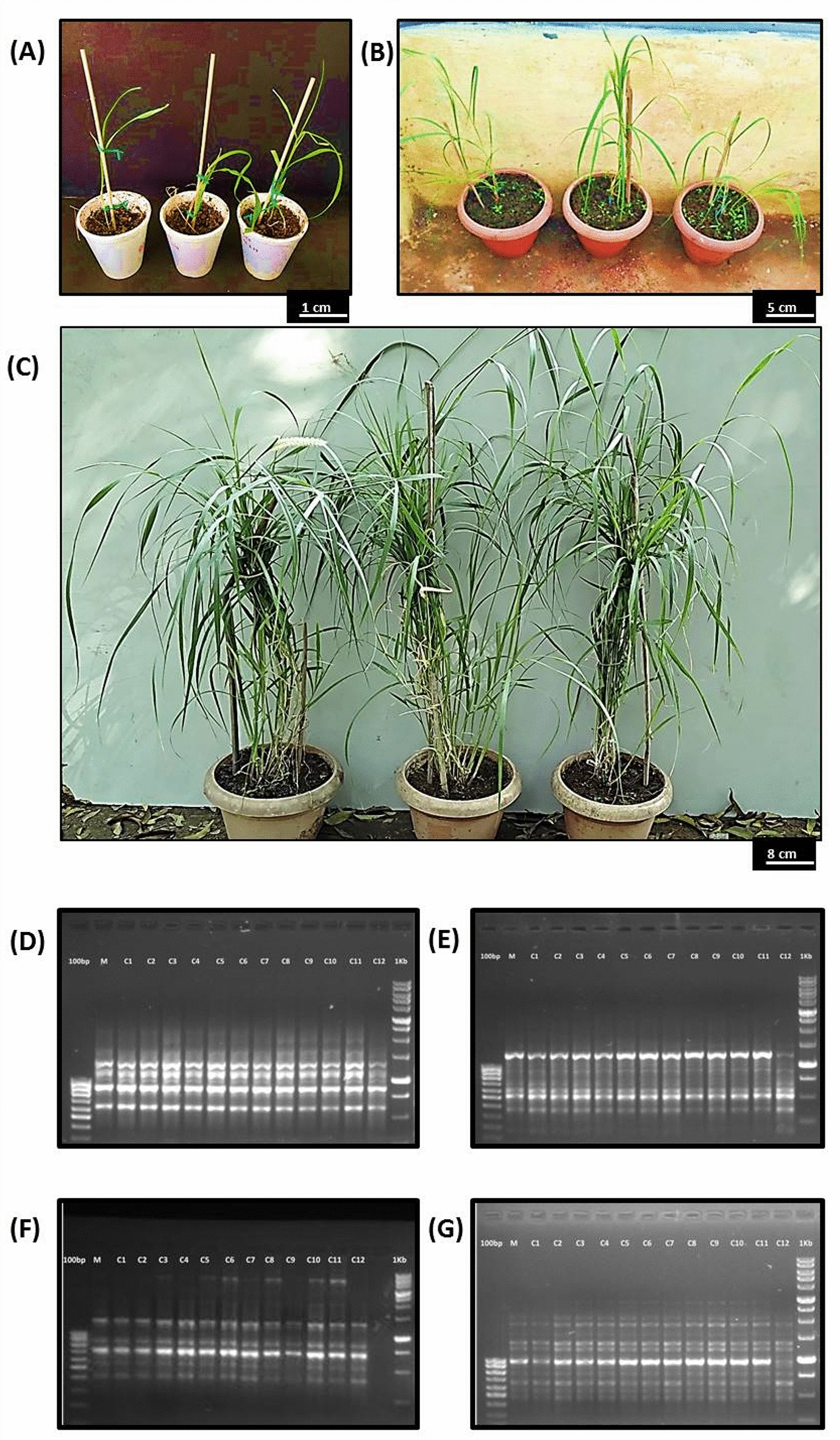
Acclimatization of regenerated plants, **A** plants growing in a growth room in thermocol cups (scale bar = 1 cm), **B** plants in garden soil kept in the greenhouse for adaptation **C** well-acclimatized plants bearing flowers in garden soil (**D**–**G**) RAPD marker profiles with four different primer sets. Lane 1 for 100 bp ladder, lane 2 for mother plant, lane 3–14 for 12 regenerated plants, lane 15 for 1 kb ladder, **A** OPD-01 primer, **B** OPB-18 primer, **C** OPK-02 primer and **D** OPA-09 primer

Plantlets obtained by in vitro culture of cell suspension passage were very soft in appearance. They were handled carefully when transferred from MS medium to Soilrite (Fig. 7A). Most of the plants survived in soil cups and were observed to be healthy after 1 week of transfer. When the plant height reached 12–18 inches, they were transferred to garden soil pots and were shifted to the greenhouse from the growth room (Fig. 7B). Sixty percent of plants could be successfully acclimatized under environmental conditions (Fig. 7C).

### Genetic fidelity

Genetic similarity with the mother plant was confirmed by PCR analysis using RAPD primers (Table 5). Eighteen primers were screened in 12 in vitro*-*raised plants along with the mother plant DNA as a control, and a uniform banding pattern was observed on the gel (Fig. 7D–G).

**Table 5:** RAPD primers used in the fidelity test

S. no.	Primer codes	Sequence (5ʹ-3ʹ)	Total no. of bands scored	Size range (in bp)
1	OPD-11	AGCGCCATTG	09	450–3000
2	OPI-16	TCTCCGCCCT	12	600–4000
3	OPB-18	CCACAGCAGT	08	75–1500
4	OPB-19	ACCCCCGAAG	07	400–3000
5	OPD-08	GTGTGCCCCA	04	700–2000
6	OPB-06	TGCTCTGCCC	07	550–4000
7	OPA-09	GGGTAACGCC	10	425–3300
8	OPC-03	GGGGGTCTTT	07	750–3500
9	OPJ-15	TGTAGCAGGG	06	625–2000
10	OPC-19	GTTGCCAGCC	07	400–1250
11	OPE-06	AAGACCCCTC	07	600–4000
12	OPA-07	GAAACGGGTG	01	500–500
13	OPA-13	CAGCACCCAC	07	800–2800
14	OPA-15	TTCCGAACCC	04	1000–2300
15	OPD-01	ACCGCGAAGG	10	600–3500
16	OPC-20	ACTTCGCCAC	08	360–1400
17	OPC-01	TTCGAGCCAG	07	220–3100
18	OPK-02	GTCTCCGCAA	06	700–7000
	Total		127	

**Fig. 8:**
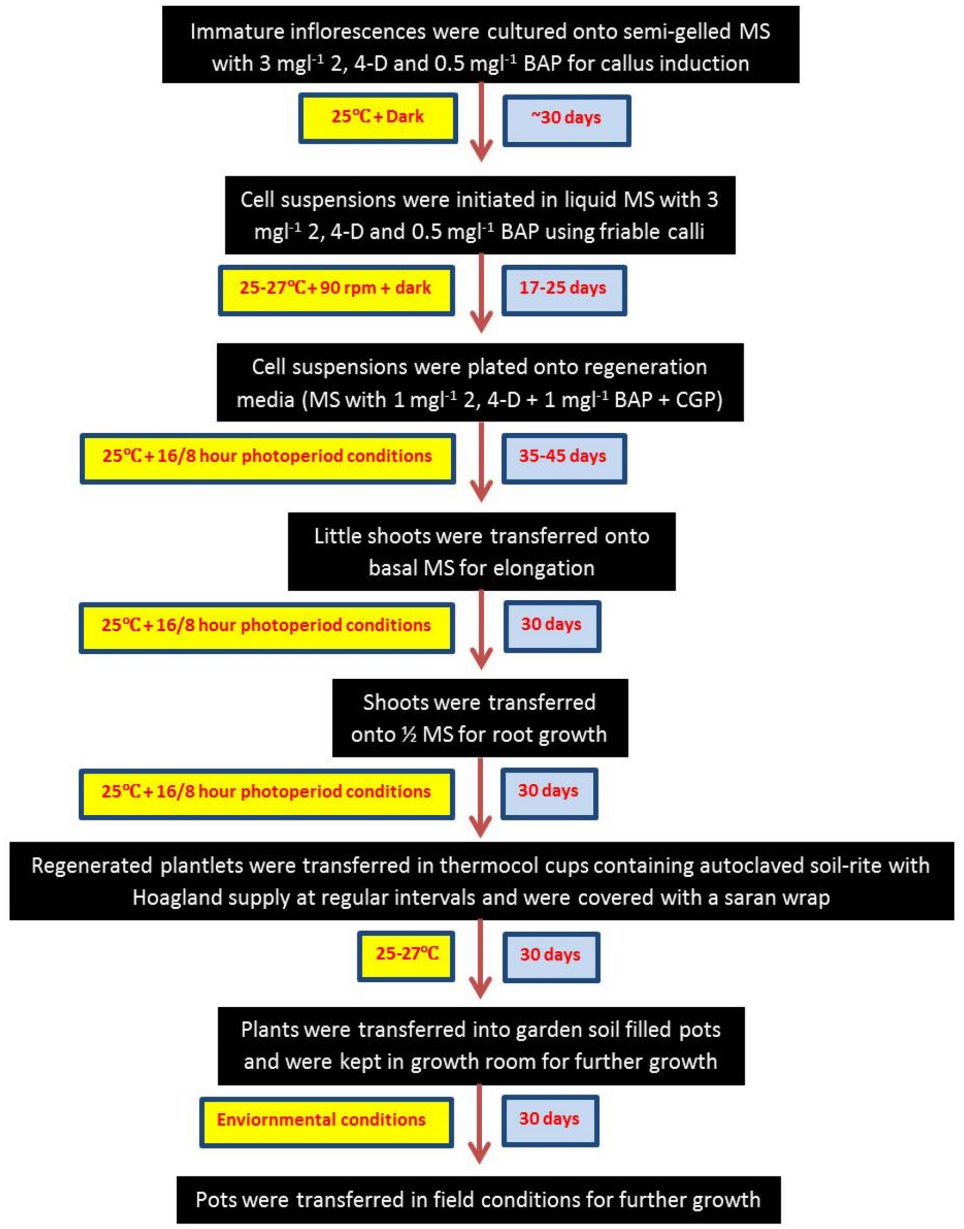
Flow chart for cell suspension establishment, regeneration and acclimatization of plants

The stepwise protocol for in vitro plant regeneration from cell suspension cultures is summarized in Fig. 8.

## Discussion

The establishment of cell suspension culture provides an excellent opportunity to induce somatic embryos, as it deals with single cells or small aggregates of cells multiplying in an agitated liquid medium. It thus accelerates the production of embryogenic cells, which are desirable for transgenic development [21]. Several attempts at in vitro plant regeneration through direct and indirect shoot organogenesis using many explants could not lead to the optimization of genetic transformation [1, 7, 8]. One of the major limitations of callus-mediated in vitro plant regeneration in *C. ciliaris* has been the loss of regeneration potential over the long-term maintenance of embryogenic calli owing to the necessity to use a higher level of cytokinins during subculturing. To overcome such major limitations, cell suspension cultures were established in the said species. In *Panicum halli*, cell suspension cultures could generate calli faster for regeneration than the calli generated on solidified media [22].

Callus induction, growth and embryogenic callus production were accomplished earlier in *C. ciliaris* using immature inflorescence explants [1, 23], especially at stage IV [8] for the cultivar IGFRI-3108. Cell suspension cultures were successfully established in liquid MS medium containing 3 mg l^−1^ 2,4-D and 0.5 mg l^−1^ BAP on shaking from friable calli induced from immature inflorescence explants. In *Glycine max* and *Trifolium repens*,* an* auxin to cytokinin ratio of 5:1 was used for cell culture initiation [24], which is very similar to the ratio that we used in cell cultures of *Cenchrus ciliaris,* i.e., 6:1.

The embryogenic cell suspensions could be maintained for a long time in the same medium, which is an improvement over our earlier studies [1, 8]. Since the use of an increased concentration of BAP during callus-mediated regeneration could have influenced the regeneration frequency of embryogenic calli during maintenance [8], embryogenic cell suspensions could indeed be maintained for a long time at a high auxin (2,4-D) concentration without the impact of aging on the regeneration potential of cell suspensions.

Optimization of inoculum density during cell suspension has a direct influence on the subculture interval. In *C. ciliaris,* an optimum inoculum size of 10 g l^−1^ was found to be best for achieving the highest cell growth, retention of viable cells and their sustainment for a longer duration, while the cell doubling time was 11 days. Based on growth curve analysis, a subculture interval of 20 days was determined to harvest cells at the end of the exponential phase. Although not much difference was noticed for the 15 g l^−1^ inoculum size at the exponential phase, a significant difference was observed for the live cell sustainment in the culture. This could be ascribed to poor ventilation of the cells due to higher cell density in the medium. As the inoculum size was increased, biomass production and viable cells in culture at the end of the growth cycle were also reduced in *Angelica sinensis* [25]. In *Pinus pinea,* the growth kinetics were determined using 20 mg ml^−1^ inoculum, where settled cell volume and dry weight peaked after 21 days, which is similar to our study where we used 10 g l^−1^ inoculum. In *Arundo donax* (perennial grass), suspension cultures were established using axillary bud explants. They observed a fivefold increase in tissue mass when DBAP media was used compared to a 1.4-fold increase in the CIM medium [26]. Thus, the growth of suspension culture was also influenced by orbiting speed, initial inoculum density, genotype, media and type of carbohydrate in the medium [27].

When the cell suspensions were transferred to MS medium containing 1 mg l^−1^ 2,4-D and 1 mg l^−1^ BAP along with some growth adjuvants, globular embryos and scutellar notches were observed. Shoot buds could be induced on the same medium. While we had previously optimized shoot induction using higher levels of cytokinins, i.e., BAP at 3 mg l^−1^ and lower levels of auxin, i.e., 2,4-D at 0.5 mg l^−1^ [1, 28], our recent experiments showed optimum levels of cytokinin: auxin at 1:1 that helped the progression and maturation of somatic embryos as well as shoot development. In another study of *Pennisetum americanum*, if the exogenously supplied auxin concentration was above the critical level required for embryogenesis, the scutellar cells were transformed back into embryogenic cells [29]. Another improvement from our earlier studies was accomplishing shoot elongation on basal MS medium without the addition of any growth hormones or plant growth regulators.

Well-developed shoots showed rooting in basal MS and ½ MS media without exogenously supplied auxin. This is in contrast to our earlier report on *C. ciliaris,* where 0.8% charcoal was used with ½ MS [23]. Other studies of rooting in *C. ciliaris* reported the addition of either 3 mg l^−1^ IAA during direct in vitro plant regeneration [30] or 2 mg l^−1^ IBA along with activated charcoal [2] for callus-mediated regeneration. The genetic fidelity experiment demonstrated that there was no soma-clonal variation.

## Conclusion

Highly reproducible cell suspension cultures were established in *C. ciliaris* in liquid MS medium containing 3 mg l^−1^ 2,4-D and 0.5 mg l^−1^ BAP using an optimized inoculum of 10 g l^−1^. A subculture interval of 20 days was determined based on the growth dynamics of cell suspensions. The inherent reduction in in vitro plant regeneration associated with prolonged subculturing of embryogenic calli has been overcome by generating relatively homogeneous cell populations while maintaining the cell suspension cultures in a high auxin-supplemented MS medium. Somatic embryos were induced in MS medium containing 1 mg l^−1^ 2,4-D and 1 mg l^−1^ BAP along with growth adjuvants followed by shoot and root development in basal and half MS, respectively. Well-developed healthy plantlets were raised, and their genetic fidelity analysis confirmed that they were true to the type of mother plant. While this novel procedure opens up new possibilities for the genetic improvement of buffelgrass, it can further be expanded to include other cultivars for many applications.

## Data Availability

All data generated and analysed during this study are included in this article.

## Abbreviations

2,4-D: 2,4-dichlorophenoxyacetic acid
BAP: 6-benzylaminopurine
TDZ: Thiadiazuron
IAA: Indole 3-acetic acid
IBA: Indole 3-butyric acid
PCV: Packed cell volume
SEM: Scanning Electron Microscopy
CPD: Critical point drying
CTAB: Cetyltrimethylammonium bromide
